# Modeling the behavior of motorcyclist with Millon and ADHD: regression modeling

**Published:** 2019-08

**Authors:** Shila Hasanzadeh, Mohammad Asghari Jafarabadi, Homyoun Sadeghi-Bazargani

**Affiliations:** ^*a*^Department of Statistics and Epidemiology, Faculty of Health, Tabriz University of Medical Sciences, Tabriz, Iran.; ^*b*^Associate Professor in Statistics and Epidemiology, Department of Statistics and Epidemiology, Faculty of Health, Tabriz University of Medical Sciences, Tabriz, Iran.; ^*c*^Associate Professor in Epidemiology, Road Traffic Injury Research Center, Tabriz University of Medical Sciences, Tabriz, Iran.

## Abstract

**Background:**

Modeling the behavior of motorcyclist with Millon and ADHD: Regression Modeling Abstract Background and Objectives: Road traffic accidents are one of the serious public health problems in the world and human factors can be effective. This study was carried on modeling of the relationship between MRBQ with Millon and ADHD.

**Methods:**

In this case-control study, 300 cases and 156 controls were selected using a cluster random sampling in Tabriz, Iran. Regression modeling to investigate the relationship between MRBQ with Millon and ADHD was studied with SPSS25 software.

**Results:**

Due to the R Square was predictable for the overall model approximately 30%, case group models 35% and for the control group was approximately 18% of MRBQ by Millon and ADHD. The standard coefficients (confidence interval) and the probable significance of the relationship between MRBQ with Millon and ADHD were obtained as follows. In generally: Millon (B= 0.17 (CI (0.95) 0.06, 0.17), P<0.001) ADHD (B= 0.48 (CI (0.95) 0.80, 1.15), P< 0.001) In Case group: Millon (B= 0.20 (CI (0.95) 0.06, 0.18), P< 0.001) ADHD (B= 0.50 (CI (0.95) 0.82, 1.15), P< 0.001) In Control group: Millon (B= 0.10 (CI (0.95) -0.04, 0.20), P=0.204) ADHD (B= 0.39 (CI (0.95) 0.44, 1.07), P< 0.001).

**Conclusions:**

The results showed, that the behavior of the people who accident (case group), There was a stronger relationship with Millon and ADHD. Therefore, planning appropriate and fits with the conditions of individuals it is necessary to accident avoidance and improved their quality of life.

**Keywords:**

MRBQ, ADHD, Linear regression, Millon

